# Single-Center In-Hospital and Outpatient Opioid Use for Lower Extremity Arterial Disease

**DOI:** 10.7759/cureus.59963

**Published:** 2024-05-09

**Authors:** Xuanjia Fan, Nicholas M Graziane, Maria Camila Castello Ramirez, Salvatore L Stella, Prasanna Karunanayaka, Victor Ruiz-Velasco, Sanjib Adhikary, Tanya Flohr

**Affiliations:** 1 Anesthesiology and Perioperative Medicine, Penn State University College of Medicine, Penn State Health Milton S. Hershey Medical Center, Hershey, USA; 2 Vascular Surgery, Penn State Heart and Vascular Institute, Penn State University College of Medicine, Penn State Health Milton S. Hershey Medical Center, Hershey, USA; 3 Neural and Behavioral Sciences, Penn State University College of Medicine, Penn State Health Milton S. Hershey Medical Center, Hershey, USA; 4 Radiology, Penn State University College of Medicine, Penn State Health Milton S. Hershey Medical Center, Hershey, USA

**Keywords:** in-patient, critical limb ischemia, peripheral artery disease, vascular intervention, opioids

## Abstract

Introduction: The pain associated with lower extremity arterial disease is difficult to treat, even with lower extremity revascularization. We sought to evaluate in-hospital and post-operative opioid usage in patients with different disease severities and treatments for lower extremity vascular disease.

Methods: A retrospective review was performed for all hospital encounters for patients with an International Classification of Diseases (ICD) code consistent with lower extremity arterial disease admitted to a single center between January 2018 and March 2023. Cases included patients admitted to the hospital with a primary diagnosis of lower extremity arterial disease. These patients were subdivided based on disease severity, treatment type, and comorbid diagnosis of diabetes mellitus. The analysis focused on in-hospital opioid use frequency and dosage among these patients. The control group (CON) included encounters for patients admitted with a secondary diagnosis of lower extremity atherosclerotic disease. A total of 438 patients represented by all the analyzed encounters were then reviewed for the number and type of vascular procedures performed as well as opioid use in the outpatient setting for one year.

Results: Critical limb ischemia (CLI) encounters were more likely to use opioids as compared to the CON and peripheral arterial disease (PAD) without rest pain, ulcer or gangrene groups (CLI 67.9% (95% CI: 63.6%-71.6%) versus CON 52.1% (95% CI: 48.5%-55.7%), *p* < 0.001 and CLI 67.9% (95% CI: 63.6%-71.6%) versus PAD 50.2% (95% CI: 42.6%-57.4%), *p* < 0.001). Opioid use was also more common in encounters for gangrene and groups treated with revascularization (REVASC) and amputation (AMP) as compared to CON (gangrene 74.5% (95% CI: 68.5%-82.1%) versus CON 52.1% (95% CI: 48.5%-55.7%), *p* < 0.01; REVASC 58.3% (95% CI: 57.3%-66.4%) versus CON 52.1% (95% CI: 48.5%-55.7%), *p* =0.01; and AMP 72.3% (95% CI: 62.1%-74.0%) versus CON 52.1% (95% CI: 48.5%-55.7%), *p* < 0.01). Significantly increased oral opioid doses per day (MME/day) were not noted for any of the investigated groups as compared to the CON. In the outpatient setting, 186 (42.5% (95% CI: 37.2%-46.4%)) patients were using opioids one month after the most recent vascular intervention. By one year, 31 (7.1% (95% CI: 1.30%-7.70%)) patients were still using opioids. No differences in opioid usage were noted for patients undergoing single versus multiple vascular interventions at one year. Patients undergoing certain vascular surgery procedures were more likely to be using opioids at one year.

Conclusion: Patients with CLI and gangrene as well as those undergoing vascular treatment have a greater frequency of opioid use during hospital encounters as compared to those patients with less severe disease and undergoing conservative management, respectively. However, these findings do not equate to higher doses of opioids used during hospitalization. Patients undergoing multiple vascular procedures are not more likely to be using opioids long-term (at one year) as compared to those patients treated with single vascular procedures.

## Introduction

Short of revascularization (REVASC), the pain associated with limb ischemia is difficult to treat [1,2]. Even with REVASC, patients are often left with augmented post-surgical pain as a result of long incisions made in unhealthy tissue and swelling associated with reperfusion [3]. Patients who are not candidates for REVASC and require amputation for limb ischemia also have unresolved pain exacerbated by phantom limb syndrome [4,5]. Patients dealing with limb ischemia are often discouraged from using nonsteroidal anti-inflammatory drugs because of the potential for worsening chronic renal insufficiency and potentiating bleeding risk [6]. Regional anesthetic options for the treatment of pain are temporary and limited [7]. These treatment constraints leave opioids as the most frequently used analgesics for ischemic limbs. Opioids, while initially efficacious, eventually lose their effectiveness requiring increasing dosages, resulting in patient tolerance and dependence [8,9].

Over the past decade, evidence suggests that patients undergoing vascular surgery are at high risk of developing opioid dependence with approximately 10% of patients using opioids beyond three months [10]. Recently, vascular surgeons sought to attribute complications from vascular surgery to prolonged opioid use and they found over 25% of patients undergoing lower extremity REVASC were still using opioids beyond six months of their original intervention with the strongest predictor of long-term use being prior opioid use [11]. Little, if any, research looks at in-hospital opioid use in patients with lower extremity vascular disease and follows those patients for continued opioid use. Given the need to minimize opioids for treating chronic pain, performing this research will help identify current in-hospital treatment strategies for patients with limb ischemia and whether alternative treatment options should be considered to reduce opioid use.

This study sought to determine (i) how frequently admission for lower extremity arterial disease is treated with opioids, (ii) how vascular disease severity affected in-hospital opioid use, and (iii) how treatment with either REVASC or amputation (AMP) as opposed to patients admitted with a secondary diagnosis of lower extremity atherosclerotic disease (controls) affect the rate of in-hospital opioid use. We hypothesized that opioid use is increased with disease severity, with a secondary diagnosis of diabetes and with vascular disease treatment as opposed to controls.

Using institutional data, the primary outcome measure of this study was to identify opioid usage during hospital encounters among patients with lower extremity arterial disease. Secondary outcomes included investigating the continuation of opioid usage, the effect of disease severity on opioid usage, and the impact of vascular treatment, including REVASC and AMP, on opioid usage.

## Materials and methods

Institutional data of in-hospital opioid use

All hospital admissions for patients with a diagnosis of lower extremity vascular disease were reviewed for Penn State Milton S. Hershey Medical Center between January 2018 and March 2023. Permission for this retrospective review was granted by the Penn State College of Medicine Institutional Review Board, IRB protocol 22745. Hospital encounters for review were identified by International Classification of Diseases (ICD) codes designating patients with lower extremity atherosclerotic disease or lower extremity diabetic peripheral angiopathy which are listed in Appendices in Supplementary Table 4. Encounters for patients with ICD codes designating atherosclerotic disease in an “other” extremity or an “unspecified” extremity were excluded from this analysis.

Encounters were further subcategorized by patient vascular disease using listed ICD diagnosis codes. Encounters for patients having occlusive peripheral arterial disease (PAD) were designated if the diagnosis code did not include rest pain, ulceration, or gangrene or if the code listed claudication. Encounters for patients were subcategorized as critical limb ischemia (CLI) if the diagnosis code listed included rest pain, ulceration, or gangrene. Encounters with CLI and PAD were further subcategorized into those with an underlying diagnosis of diabetes mellitus (DM). Separating patients with PAD and critical limb ischemia CLI based on DM diagnosis allows for the examination of opioid use patterns in more homogeneous subgroups, considering potential differences in pain profiles, treatment strategies, and clinical outcomes.

Encounters were also subcategorized by intervention during hospital admission. Patients admitted with a secondary diagnosis of lower extremity atherosclerotic disease were control subjects (CON). These encounters included patients with a vascular diagnosis admitted for reasons other than vascular disease. The encounter was categorized as being for REVASC if the admitted patient had a current procedure terminology (CPT) code designating a REVASC procedure. These procedures included angiogram +/- angioplasty, stenting, embolectomy, aortobifemoral or aortobi-iliac bypass, endarterectomy, and lower extremity bypass. The encounter was categorized as being for major AMP if an associated CPT code was designated below or above knee AMP. Encounters for patients undergoing isolated toe or transmetatarsal AMPs during hospitalization were grouped with the REVASC group. Vascular surgery CPT codes identified and associated with CON, REVASC, and AMP groups are listed in Appendices in Supplementary Table 5.

CON was compared to patients admitted for PAD and to patients admitted for CLI. Groups were compared based on opioid usage and the amount used during hospitalization. Encounters by vascular disease ICD code designation (as having claudication, rest pain, gangrene, or isolated atherosclerotic disease without the aforementioned complaints) were evaluated for opioid use and the amount used during hospitalization. CON was compared to encounters representing patients undergoing REVASC and encounters representing patients undergoing AMP for opioid usage and amount during hospitalization.

Demographics assessed for the aforementioned groups include average age, male gender, and average days admitted during the encounter. Hospitalization encounters were considered positive for opioids if the following medications were listed as being given during the admission: hydromorphone, morphine, methadone, meperidine, fentanyl, oxycodone, hydrocodone, and codeine. Encounters with opioids given only at the time of surgical intervention were not considered positive for opioids. Average daily morphine milligram equivalents were calculated for the encounters.

Institutional data of outpatient opioid use

Electronic medical records for individuals with PAD and CLI having multiple hospital admissions during the selected time period were reviewed. Patient demographics including age (as of January 2018), gender, and most recent survival status were collected. The vascular surgical interventions were categorized as angiogram +/- angioplasty, stenting, embolectomy, aorto-bi-femoral or aorto-bi-iliac bypass, endarterectomy, lower extremity bypass, toe AMP, transmetatarsal AMP, below knee AMP or above knee AMP. Patients were categorized as REVASC or AMP.

In order to better evaluate the number of vascular interventions contributing to long-term opioid usage, patients with multiple vascular interventions during the period of inquiry were evaluated. The date of the patient’s last vascular surgical intervention was noted, and subsequent outpatient surgical evaluations were reviewed for patient narcotic usage. Patients having narcotics listed on their outpatient surgical records beyond one month after their last vascular surgical intervention were considered chronic narcotic users. If the patient did not have narcotics listed on their outpatient surgical records beyond one month, they were considered not chronic narcotic users. For those patients with narcotics listed greater than one month beyond their last vascular surgery date, the number of months positive for narcotics use was recorded. For example, patients with outpatient surgical records for one month and three months post vascular surgery were considered to be using narcotics chronically for three months, even though no outpatient surgical records for two months were available to review. If the same patient was seen five months post vascular surgery and no narcotics were listed on the fifth-month outpatient records, the patient was considered a chronic narcotic user for three months only.

Outcome measures

Primary Outcome

The primary outcome measure of this study was to identify opioid usage during in-hospital encounters among patients with lower extremity arterial disease.

Secondary Outcomes

Secondary outcomes included investigating the continuation of opioid usage post-discharge. Secondly, the study assessed the effect of disease severity on opioid usage by subcategorizing encounters based on clinical parameters. Finally, the impact of vascular treatment modalities, including REVASC and AMP, on opioid usage was evaluated.

Statistical analysis

This study adhered to the STROBE (STrengthening the Reporting of OBservational studies in Epidemiology) guidelines for reporting observational studies. The checklist of items recommended by STROBE was used to ensure comprehensive and transparent reporting of the study methods, results, and interpretation. In this study, a convenient sampling approach was employed to select participants. Due to practical constraints and the retrospective nature of the study, a predefined sample size calculation was not performed. Instead, patients meeting the inclusion criteria during the study period were included in the analysis. In our analysis, we categorized the patients into various groups based on predefined criteria. This approach yielded an uneven number of patients across groups. However, no adjustments were attempted to account for these imbalances in the analysis. All variables were summarized with frequencies and percentages or medians with interquartile ranges (IQR). Variables were compared using Wilcoxon and chi-squared tests for continuous and categorical variables, respectively. Pearson’s correlation coefficient was used to determine the correlation between continuous variables and reported with r values. The Wilson score interval method was used to calculate 95% confidence intervals for binary data sets. Statistical significance was set at a p-value of < 0.05. Analyses were performed using R version 4.3.2 Statistical Package (Released 2023; R statistical software (R Foundation for Statistical Computing, Vienna, Austria) or GraphPad Prism Software Version 10.0.2 (GraphPad Software, San Diego, CA).

## Results

In-hospital opioid use for vascular encounters

A total of 1359 in-patient hospital encounters (1043 patients) were identified during the five-year period (January 2018-March 2023). Demographics for CON, vascular disease severity (PAD and CLI), vascular signs/symptoms on presentation (claudication, rest pain, ulcer, and gangrene), and vascular treatment groups (REVASC and AMP) are listed in Table 1. Ages for each group were similar to CON with the exception of REVASC encounters (CON: 69 (62-77) years; REVASC: 67 (60-74); p < 0.01, Wilcoxon test) (Table 1). Sex distribution was similar for all groups of encounters as compared to CON (PAD vs. CON: c2 = 0.0005, df =1; CLI vs CON: c2 = 0.07, df =1; chi-squared test) (Table 1). PAD, claudication, rest pain, and REVASC encounters had shorter admissions as compared to CON (PAD vs. CON: W = 125082, p < 0.001; Claudication vs. CON: W = 38593, p < 0.01; Rest pain vs CON: W = 45795, p < 0.01; REVASC vs. CON: W = 164888, p = 0.01; Wilcoxon test) (Table 1). Gangrene and AMP encounters had longer admissions as compared to CON (Gangrene vs. CON: W = 56814, p = 0.03; AMP vs. CON: W = 69420, p < 0.01; Wilcoxon test) (Table 1).

**Table 1:** Institutional data separated by vascular disease severity, vascular signs/symptoms on presentation, and vascular treatment. Percentage of hospital admissions positive for opioids noted across “+ opioid” row. p-values as compared to CON. *p < 0.05; **p < 0.001. LOS: Length of stay; IQR: interquartile range; CON: control; PAD: peripheral arterial disease; CLI: critical limb ischemia; REVASC: revascularization; AMP: amputation

		Vascular Disease Severity		Vascular Signs/Symptoms on Presentation				Vascular Treatment	
Group n (%)	CON 793 (58.4%)	PAD 242 (17.8%)	CLI 324 (23.8%)	Claudication 64 (5.5%)	Rest pain 97 (8.4%)	Ulcer 41 (3.54%)	Gangrene 161 (13.9%)	REVASC 379 (26.8%)	AMP 242 (17.1%)
Age in years Median (IQR) p-value	69 (62-77)	69.5 (62-75) 0.37	68 (61-77) 0.5	68 (63-72) 0.38	66 (61-74) 0.11	69 (62-80) 0.32	67 (61-77) 0.45	67 (60-74)* <0.01	68 (61-76) 0.12
Male Gender n (%) p-value	515 (64.9%)	158 (65.3%) 0.98	207 (63.8%) 0.79	45 (70.3%) 0.32	63 (64.9%) 1	24 (58.5%) 0.5	102 (63.4%) 0.77	245 (64.6%) 0.21	162 (66.9%) 0.62
LOS in days Mean (IQR) p-value	7.0 (4.0-12.0)	4.5 (3.1-6.8)** < 0.001	7.0 (4.2-11.3) 0.87	3.3 (2.5-4.9)* <0.01	5.3 (3.3-8.2)* <0.01	6.5 (4.3-9.2) 0.67	8.3 (4.3-14.7)* 0.03	6.0 (4.2-9.2)* 0.01	9.8 (6.4-15.9)* <0.01
+ Opioid n (%) p-value	413 (52.1%)	121 (50.2%) 0.62	220 (67.9%)** <0 .001	27 (42.2%) 0.16	59 (60.8%) 0.13	24 (58.5%) 0.5	120 (74.5%)* <0.01	221 (58.3%)* 0.048	175 (72.3%)* <0.01
MME/d Opioid Mean (IQR) p-value	4.4 (2-14.8)	4.8 (1.9-16.4) 0.79	5.1 (2-14) 0.39	6.3 (2-14.8) 0.52	4.6 (3-13.1) 0.35	4.3 (1.5-11.6) 0.68	5.9 (1.9-16.9) 0.6	5.4 (2.2-16.1) 0.24	5.2 (2-16.3) 0.06
+ Acetaminophen n (%) p-value	102 (12.9%)	22 (9.1%) 0.14	39 (12%) 0.78	4 (6.3%) 0.18	10 (10.3%) 0.58	6 (14.6%) 0.93	20 (12.4%) 0.98	47 (12.4%) 0.9	36 (14.9%) 0.48

In examining opioid use, we found that CLI encounters were more likely to use opioids as compared to the CON group (CLI 67.9% (95% CI: 63.6%-71.6%) versus CON 52.1% (95% CI: 48.5%-55.7%); c2 = 22.8 df = 1; p <0.001; chi-squared test) (Table 1). Additionally, opioid use was more common in gangrene encounters and both vascular treatment groups (REVASC and AMP) as compared to CON (Gangrene vs. CON: c2 = 26.5 df = 1, p < 0.01; REVASC vs. CON: c2 = 15.9 df = 1, p = 0.048; AMP vs. CON: c2 = 30.1 df = 1, p < 0.01; chi-squared test) (Table 1). We found no significant differences in oral opioid doses per day (MME/d) calculated for any of the investigated groups as compared to CON (Table 1).

Given that acetaminophen is often used as a non-opioid analgesic option for patients undergoing surgical procedures [12], we investigated the in-hospital use of acetaminophen between groups. We found that all groups used acetaminophen at similar rates compared to CON with no significant difference in the use of opioids versus acetaminophen across the nine groups (c2 = 5.409, df = 8, p = 0.7131; chi-square test) (Table 1). These results indicate that individuals in one group were not more likely to be given acetaminophen compared to individuals in another group.

To examine in-patient opioid use patterns in subjects with comorbid DM, PAD and CLI encounters were separated by a diagnosis of DM, and demographics for the groups were evaluated. No difference in age or gender was noted between the PAD or CLI groups with or without a diagnosis of DM (PAD comparison c2 = 0.87 df = 1, CLI comparison c2 = 0.44 df = 1; chi-squared test) (Table 2). Both PAD +DM and CLI +DM encounters were found to have significantly longer hospital admissions as compared to their respective -DM groups (PAD comparison: W = 3853, p < 0.01; CLI comparison: W = 9548, p < 0.01; Wilcoxon test) (Table 2). The frequency of narcotic use, MME/d and the frequency of acetaminophen use were not found to be significantly different with a diagnosis of DM (PAD comparison: c2 = 3.46 df =1, CLI comparison: c2 = 0.94 df = 1; chi-squared test) (Table 2).

**Table 2:** Institutional data separated by a diagnosis of DM and vascular disease severity (PAD and CLI). Percentage of hospital admissions positive for opioids noted across “+ opioid” row.  *p < 0.05. IQR: Interquartile range; PAD-DM: peripheral arterial disease minus diabetes mellitus; PAD+DM: peripheral arterial disease plus diabetes mellitus; CLI - DM: critical limb ischemia minus diabetes mellitus; CLI + DM: critical limb ischemia plus diabetes mellitus

Group n (%)	PAD-DM 68 (28.1%)	PAD+DM 174 (71.9%)	CLI-DM 179 (55.2%)	CLI+DM 145 (44.8)
Age in years Median (IQR) p-value	69.5 (63-74)	69.5 (62-75) 0.82	68 (62-77)	67 (60-77) 0.40
Male Gender n (%) p-value	48 (70.6%)	110 (63.2) 0.35	111 (62%)	96 (66.2%) 0.51
LOS in days Median (IQR) p-value	3.4 (2.5-4.9)	5.3 (3.3-7.5)* <0.01	6 (3.7-9.1)	8.9 (4.9-15.0)* <0.01
+ Opioid n (%) p-value	27 (39.7%)	94 (54.0%) 0.06	117 (65.4%)	103 (71.0%) 0.33
MME/d Opioid Median (IQR) p-value	6.3 (2-14.8)	4.6 (1.9-15.5) 0.65	5.9 (2.9-15.8)	4.6 (1.5-16) 0.11
+ Acetaminophen n (%) p-value	4 (5.9%)	18 (10.3%) 0.4	19 (10.6%)	20 (13.8%) 0.48

PAD +/-DM and CLI +/-DM were further separated by vascular treatment (REVASC or AMP) and the narcotic use frequency and MME/d were evaluated. Of note, 26 PAD-DM, 56 PAD +DM, 52 CLI -DM, and 36 CLI +DM encounters underwent no intervention (Table 3). We found that patients with DM undergoing REVASC did not use narcotics more frequently than their respective -DM groups (PAD REVASC comparison: c2 = 0.0253 df =1; CLI REVASC comparison: c2 = 0.6808 df =1; chi-squared test) (Table 3). Additionally, no difference in MME/d was noted for PAD +DM and PAD -DM encounters undergoing REVASC (W = 506; Wilcoxon test) and no difference in MME/d was noted for CLI +MD and CLI -DM encounters undergoing REVASC (W = 504; Wilcoxon test) (Table 3). There was no significant difference in narcotic use frequency between CLI +DM and CLI -DM encounters undergoing AMP (CLI AMP comparison: c2 = 0.24 df =1; chi-squared test) (Table 3). MME/d calculated for the CLI +DM encounters undergoing AMP was significantly less than the MME/d calculated for CLI -DM encounters undergoing AMP (CLI REVASC comparison: W = 629, p = 0.01; Wilcoxon test) (Table 3). 

**Table 3:** Opioid use for vascular procedure treatment groups (REVASC and AMP) separated by a diagnosis of DM and vascular disease severity. Percentage of hospital admissions positive for opioids noted across “+ opioid” rows. *p < 0.05. IQR: Interquartile range; REVASC: revascularization; AMP: amputation; PAD-DM: peripheral arterial disease minus diabetes mellitus; PAD+DM: peripheral arterial disease plus diabetes mellitus; CLI - DM: critical limb ischemia minus diabetes mellitus; CLI + DM: critical limb ischemia plus diabetes mellitus

Group n (%)	PAD-DM 68 (28.1%)	PAD+DM 174 (71.9%)	CLI-DM 179 (55.2%)	CLI+DM 145 (44.8)
REVASC	n=42	n=103	n=91	n=23
+ Opioid n (%) p-value	21 (50%)	50 (48.5%) 1	59 (64.8%)	17 (73.9%) .56
MME/d Opioid Mean (IQR) p-value	3.9 (1.8-12.4)	5.3 (1.8-17.3) 0.82	5.8 (2.3-19.0)	8.1 (1.5-14.4) 0.98
AMP	n=0	n=15	n=36	n=86
+ Opioid n (%) p-value	0	10 (66.7%) -	29 (80.6%)	64 (74.4%) 0.62
MME/d Opioid Mean (IQR) p-value	-	9.6 (3.2-16.1) -	9.1 (4.2-27.6)	4.2 (1.9-14.1)* 0.01

Outpatient opioid use

During the five-year period, a total of 438 patients (representing 242 PAD and 324 CLI encounters) were admitted to the hospital with a primary diagnosis of either PAD or CLI and retrospectively reviewed for outpatient opioid use. At one month after the most recent lower extremity vascular surgery intervention for REVASC or AMP, 238 (54.3% (95% CI: 50.7%-60.5%)) were no longer taking opioids and 186 (42.5% (95% CI: 37.2%-46.4%)) continued to take opioids. A total of 14 (3.2% (95% CI: 0.60%-2.80%)) patients had no follow-up to assess opioid use. The most common opioid prescribed was oxycodone followed by hydrocodone-acetaminophen and hydromorphone. For those using opioids greater than one month, the median use time was three months (IQR 3-8 months). Six months after the most recent lower extremity vascular intervention, 54 (12.3% (95% CI: 10.3%-17.4%)) patients were still using opioids. By twelve months, 31 (7.1% (95% CI: 1.30%-7.70%)) patients were still using opioids. By the end of the study, 108 of the 438 (24.6% (95% CI: 22.5%-30.4%)) patients treated during the five-year study period were deceased. There were no differences in the number of deceased patients taking narcotics and not taking narcotics (p =0.38; chi-squared test).

Post-intervention outpatient opioid use

During a five-year period, 81 of 438 patients (18.5% (95% CI: 17.2%-25.0%)) were noted to have multiple encounters for lower extremity vascular treatments. These 81 patients underwent a total of 345 lifetime vascular procedures with a median of four admissions per patient. Opioid use for these 81 patients was evaluated beyond their most recent hospital admission for vascular intervention. A total of 49 (60.5% (95% CI: 52.6%-73.0%)) patients were found to be using opioids beyond one month after vascular intervention. 45 of the 49 patients still using opioids had outpatient records available for evaluation beyond one month. These 45 patients used opioids for a median of four months (IQR 2-8 months). Nine (11% (95% CI: 7.30%-19.0%)) patients were using opioids beyond one year of their last vascular surgery. A total of 21 (25.9% (95% CI: 20.0%-36.9%)) patients died during the study period with no overt deaths attributable to opioid overdose. There was no statistical significance in the number of deaths for patients using opioids beyond one month after their last vascular surgery as compared to the number of deaths for patients not using opioids (12 (14.8% (95% CI: 8.80%-22.3%)) versus 8 (9.9% (95% CI: 5.10%-18.3%)), p = 0.98; chi-squared test). Of note, 10 of the 81 (12.3% (95% CI: 7.10%-19.5%)) patients were transitioned to tramadol and taking it beyond one month of the most recent vascular intervention. Three patients on tramadol (33%) were deceased at the end of the study.

**Figure 1:**
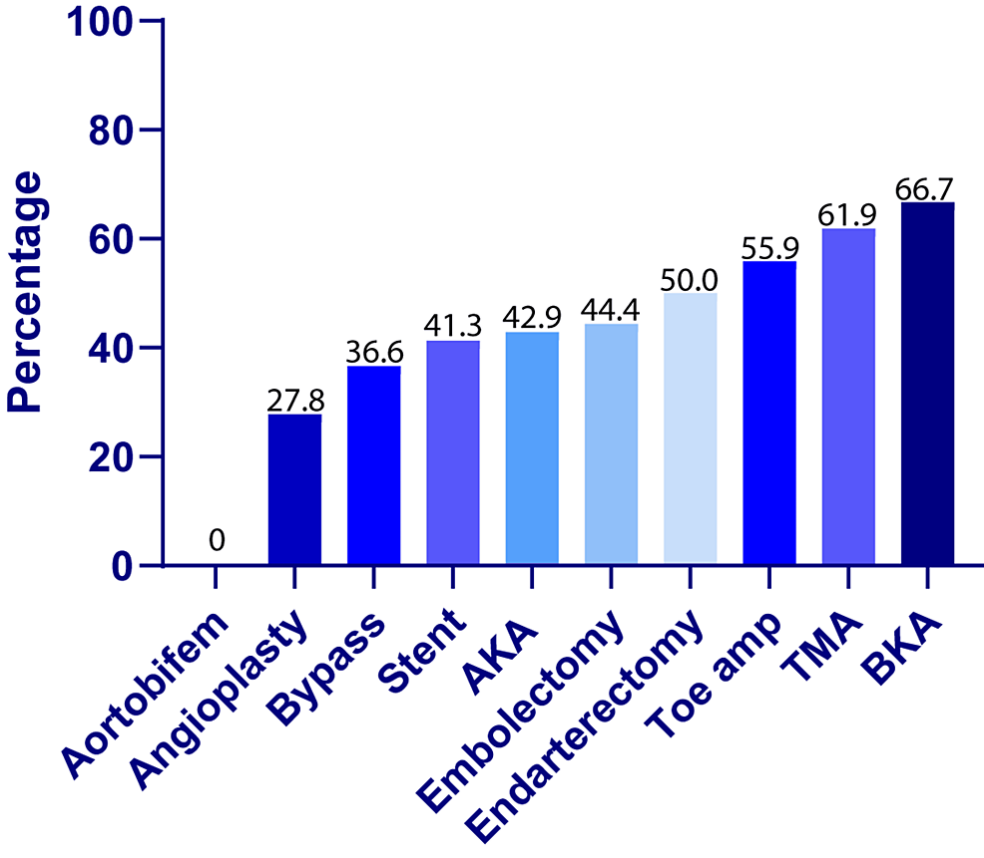
The percentage of patients having undergone each procedure type still using opioids beyond one month of the last vascular surgery. Corresponding p-values depicted at the base of the bars. Aortobifem: Aortobifemoral bypass surgery; Bypass: peripheral artery bypass surgery; Stent: peripheral artery stent placement; AKA: above knee amputation; TMA: transmetatarsal amputation; BKA: below knee amputation.

We next examined the association between vascular procedures and opioid use beyond one month of the procedure. A chi-square test was conducted to analyze the relationship between the type of vascular procedure performed and opioid use rates. The analysis included data from 45 patients who underwent 10 different vascular procedures and who had outpatient records available. Our results show a statistically significant association between vascular procedure type and opioid use rates (c2 = 59.89, df = 9, p < 0.0001; chi-square test) (Figure 1). These findings indicate that the likelihood of opioid use varied significantly depending upon the type of vascular procedure performed. However, it is important to note that these results may also be influenced by the number of procedures undergone per patient.

Finally, we compared patients taking opioids beyond one month of the last vascular intervention to those not taking opioid for total number of vascular procedures performed. Patients continuing to take opioids were not more likely to have more procedures performed as compared to those patients no longer taking opioids (3.9 procedures versus 4.1 procedures, p = 0.74; chi-squared test). For the patients continuing to take opioids beyond one month of the last vascular intervention, no correlation was observed between the number of vascular surgeries performed on the patient and the number of months reported for opioid use (r = -0.0052, p = 0.97; Pearson’s correlation coefficient).

## Discussion

This institutional retrospective review of opioid use in patients affected with lower extremity arterial disease suggests that for in-hospital encounters with increasing disease severity (CLI versus PAD, CLI versus CON, and gangrene versus CON group), the frequency of opioid use was increased. When evaluating lower extremity disease severity as associated with specific signs/symptoms (i.e., claudication, rest pain, ulceration, and gangrene), this study did not find sequential increases in the frequency of opioid use with progressive disease until gangrene was present. Notably, the amount of opioid used to treat patients with CLI and gangrene was not significantly greater. There was an increased frequency of opioid use for hospital encounters to treat lower extremity arterial disease (REVASC and AMP) as compared to the CON group. However, the amount of opioid used was not significantly greater. These findings suggest that patients with CLI, gangrene, and or undergoing a vascular procedure (REVASC and AMP) are not exposed to increased amounts of opioid during their hospitalization. A diagnosis of DM did not seem to affect the frequency of narcotic usage, however, encounters for CLI +DM patients undergoing AMP received significantly smaller doses of opioids at the same frequency. This finding can likely be attributed to a sicker, more fragile patient population for which limited opioid dose is preferred to maintain hemodynamic stability during the perioperative period. Moreover, the rate of acetaminophen use for all these populations was uniformly low as compared to the rate of opioid use.

Outpatient opioid usage for all patients evaluated, undergoing single and multiple interventions, at one month and one year was 42.5% and 7.1%, respectively. Approximately 60% of patients undergoing multiple vascular interventions were found to be using opioids beyond one month of their most recent vascular surgery intervention which was significantly increased as compared to those undergoing single vascular procedures. Only 11% of these patients having undergone multiple vascular procedures were still using opioids at one year and there was no statistical significance as compared to the number of patients treated with a single vascular intervention taking opioids one year out. These findings suggest that the frequency of opioid usage in the outpatient post-operative period after multiple vascular interventions might be increased initially, but the difference was not maintained beyond one year.

While one-quarter of the patients undergoing multiple vascular procedures were deceased at the end of the study, none of these patients were deceased from opioid overdose. Patients undergoing multiple procedures were more likely to be using opioids for more than one month after recent vascular surgery intervention. Patients having undergone multiple vascular procedures and using opioids beyond one month of their most recent vascular surgery intervention were not found to have an increased number of vascular surgery procedures performed as compared to other patients having undergone multiple vascular procedures and not using opioids. This suggests that the increased procedure number does not necessarily render the patient more susceptible to opioid dependence. No correlation between vascular procedure number and number of months of opioid use could be shown.

Similar to the study by Itog et al., our findings suggest that increased lower extremity arterial disease severity with progression to CLI was associated with more frequent opioid use during the hospital encounter, however, such patients were not treated with higher amounts of opioid [13]. As suggested by Itoga et al., the frequency of opioid use during hospital encounters increases with REVASC. AMP also showed an increased rate of opioid usage during hospital encounters, even as compared to REVASC. Because of data limitations, we were not able to examine opioid use frequency or dose amounts in patients admitted for multiple hospital encounters.

Additionally, we found that the rate of persistent opioid use (beyond one month) in patients undergoing multiple vascular procedures was comparable to previous reports that investigated chronic opioid use history in patients undergoing vascular interventions within the one to three months post-operative period [14,15]. Notably, half of the patients treated with an entirely endovascular approach were persistent opioid users showing that endovascular-only revascularizations are not the answer for reducing chronic narcotic use in this patient population [16]. This study also shows that many of those patients can be weaned successfully off opioids by one year after the most recent vascular procedure with long-term opioid prevalence similar to other surgical specialties [10]. Even though patients undergoing multiple vascular surgery interventions do not appear to have a predilection for developing opioid addiction, vascular surgery prescribers should be aware that there are some procedures that are more likely to result in long-term opioid use. Interestingly, others have suggested that prescribers are often sending patients home with prescriptions that are for higher amounts of narcotics per day and do not reflect the actual opioid usage during hospitalization [17]. Based on encounter dosing, our study suggests that patients should not be receiving more than 5 mg of oxycodone with 2-3 tablets per day at the time of discharge. We could find no studies suggesting benefit or lack of benefit in treating PAD at any severity with acetaminophen.

Study limitations include single hospital/institution data with limited numbers representing a limited patient population and a limited surgical team. Additionally, a power calculation was not performed, which may have affected the study's ability to detect significant associations or differences. Another limitation stems from the lack of outpatient medication data before hospitalization, precluding the assessment of opioid usage for non-surgical conditions that may have been ongoing prior to admission. However, our study's primary objective was to capture baseline opioid usage among patients with underlying lower extremity arterial disease during hospital encounters. We did not specifically aim to characterize opioid-naive patients who were initiated on opioids in the hospital. Our analysis also revealed that ICD codes applied to represent disease severity might not be the most accurate as patients with PAD, without CLI, were given CPT codes designating they underwent amputation. With numerous ICD codes to describe the patient presentation, vascular surgeons should attempt to be as specific as possible with diagnosis.

Finally, a better understanding of the pain associated with vascular ischemia is desperately needed. While revascularization, if possible, is the most important tenet of vascular surgery treatment, patients will suffer pain until an accurate diagnosis is made, and appropriate treatment can be performed as well as initially in the post-operative period. Focus within the vascular surgery community should be directed at developing pain medications specifically foiling the ischemic response.

## Conclusions

In conclusion, we find that patients with CLI and gangrene as well as those undergoing vascular treatment have a greater frequency of opioid use during hospital encounters as compared to those patients with less severe disease and undergoing conservative management, respectively. However, these findings do not equate to higher doses of opioid used during hospitalization. These data suggest that healthcare providers are opting for smaller, more frequent doses of opioid to maintain a consistent level of pain relief, while minimizing the risk of adverse side effects, including respiratory depression. Finally, we find that patients undergoing multiple vascular procedures are not more likely to be using opioid long-term (at one year) as compared to those patients treated with single vascular procedures.

## Appendices

**Table 4:** ICD codes designating patients with lower extremity atherosclerotic disease or lower extremity diabetic peripheral angiopathy included (columns listed PAD codes and CLI codes) and ICD codes excluded for upper extremity atherosclerotic disease or unspecified. PAD: Peripheral artery disease; CLI: critical limb ischemia

PAD Codes	CLI Codes		Codes Excluded for Unspecified Limb or Arm
I70.2		I70.22	I70.208
I70.20		I70.221	I70.209
I70.201		I70.222	I70.218
I70.202		I70.223	I70.219
I70.203		I70.23	I70.228
I70.21		I70.231	I70.229
I70.211		I70.232	I70.25
I70.212		I70.233	I70.268
I70.213		I70.234	I70.269
I70.29		I70.235	I70.298
I70.291		I70.238	I70.299
I70.292		I70.239	I70.308
I70.293		I70.24	I70.309
I70.3		I70.241	I70.318
I70.30		I70.242	I70.319
I70.301		I70.243	I70.328
I70.302		I70.244	I70.329
I70.303		I70.245	I70.35
I70.31		I70.248	I70.368
I70.311		I70.249	I70.369
I70.312		I70.26	I70.398
I70.313		I70.261	I70.399
I70.39		I70.262	I70.408
I70.391		I70.263	I70.409
I70.392		I70.32	I70.418
I70.393		I70.321	I70.419
I70.40		I70.322	I70.428
I70.401		I70.323	I70.429
I70.402		I70.33	I70.45
I70.403		I70.331	I70.468
I70.41		I70.332	I70.469
I70.411		I70.333	I70.498
I70.412		I70.334	I70.499
I70.413		I70.335	I70.508
I70.49		I70.338	I70.509
I70.491		I70.339	I70.518
I70.492		I70.34	I70.519
I70.493		I70.341	I70.528
I70.50		I70.342	I70.529
I70.501		I70.343	I70.55
I70.502		I70.344	I70.568
I70.503		I70.345	I70.569
I70.51		I70.348	I70.598
I70.511		I70.349	I70.599
I70.512		I70.36	I70.608
I70.513		I70.361	I70.609
I70.59		I70.362	I70.618
I70.591		I70.363	I70.619
I70.592		I70.42	I70.628
I70.593		E11. 52	I70.629
I70.6		I70.421	I70.65
I70.60		I70.422	I70.668
I70.601		I70.423	I70.669
I70.602		I70.43	I70.698
I70.603		I70.431	I70.699
I70.61		I70.432	I70.708
I70.611		I70.433	I70.709
i70.612		I70.434	I70.718
I70.613		I70.435	I70.719
I70.69		I70.438	I70.728
I70.691		I70.439	I70.729
I70.692		I70.44	I70.75
I70.693		I70.441	I70.768
I70.7		I70.442	I70.769
I70.70		I70.443	I70.798
I70.701		I70.444	I70.799
I70.702		I70.445	
I70.703		I70.448	
I70.71		I70.449	
I70.711		I70.46	
I70.712		I70.461	
I70.713		I70.462	
I70.79		I70.463	
I70.791		I70.52	
I70.792		I70.521	
I70.793		I70.522	
I70.8		I70.523	
I70.9		I70.53	
I70.90		I70.531	
I70.91		I70.532	
I70.92		I70.533	
I73.9		I70.534	
I73.8		I70.535	
I73.89		I70.538	
Z86.79		I70.539	
E08. 51		I70.54	
E08. 59		I70.541	
E09. 51		I70.542	
E09. 59		I70.543	
E10. 51		I70.544	
E10. 59		I70.545	
E11. 51		I70.548	
E11. 59		I70.549	
E13. 51		I70.56	
E13. 59		I70.561	
		I70.562	
		I70.563	
		I70.62	
		I70.621	
		E13. 52	
		I70.622	
		I70.623	
		I70.63	
		I70.631	
		I70.632	
		I70.633	
		I70.634	
		I70.635	
		I70.638	
		I70.639	
		I70.64	
		I70.641	
		I70.642	
		I70.643	
		I70.644	
		I70.645	
		I70.648	
		I70.649	
		I70.66	
		I70.661	
		I70.662	
		I70.663	
		I70.72	
		I70.721	
		I70.722	
		I70.723	
		I70.73	
		I70.731	
		I70.732	
		I70.733	
		I70.734	
		I70.735	
		I70.738	
		I70.739	
		I70.74	
		I70.741	
		I70.742	
		I70.743	
		I70.744	
		I70.745	
		I70.748	
		I70.749	
		I70.76	
		I70.761	
		I70.762	
		I70.763	
		E08. 52	
		E09. 52	
		E10. 52	

**Table 5:** CPT codes designating diagnostic imaging procedures for CON, procedures performed for REVASC, and procedures performed for AMP. CON: Control; REVASC: revascularization; AMP: amputation

CON	REVASC
75630	35521
75710	35533
75716	35541
75635	35546
	35548
	35549
	35551
	35558
	35563
	35565
	35582
	35621
	35623
	37201
	35641
	35646
	35651
	35654
	35661
	35663
	35665
	35556
	35566
	35571
	35583
	35585
	35587
	37209
	35656
	35666
	35671
	35875
	35876
	35452
	35454
	35456
	35459
	35470
	35742
	35743
	35474
	37205
	37206
	37207
	37208
	35331
	35351
	35355
	35361
	35363
	35371
	35372
	35381
	34201
	34203
